# Cross-talk between necroptosis-related lncRNAs to construct a novel signature and predict the immune landscape of lung adenocarcinoma patients

**DOI:** 10.3389/fgene.2022.966896

**Published:** 2022-09-15

**Authors:** Jie Wu, Dingli Song, Guang Zhao, Sisi Chen, Hong Ren, Boxiang Zhang

**Affiliations:** ^1^ Department of Thoracic Surgery, The First Affiliated Hospital of Xi’an Jiaotong University, Xi’an, China; ^2^ Department of Oncology, The Second Affiliated Hospital of Xi’an Jiaotong University, Xi’an, China

**Keywords:** lung adenocarcinoma, necroptosis, lncRNA, gene signature, immune landscape

## Abstract

**Background:** As a new style of cell death, necroptosis plays a crucial role in tumor immune microenvironment. LncRNAs have been identified to act as competitive RNAs to influence genes involved in necroptosis. Therefore, we aim to create a signature based on necroptosis-related lncRNAs to predict the prognosis and immune landscape of lung adenocarcinoma (LUAD) patients in this study.

**Methods:** TCGA database was used to acquire RNA sequencing (RNA-Seq) data and clinical information for 59 lung normal samples and 535 lung adenocarcinoma samples. The Pearson correlation analysis, univariate cox regression analysis and least absolute shrinkage and selection operator (LASSO) cox regression were performed to construct the prognostic NRlncRNAs signature. Then we used Kaplan-Meier (K-M) analysis, time-dependent ROC curves, univariate and multivariate cox regression analysis, and nomogram to validate this signature. In addition, GO, KEGG, and GSVA were analyzed to investigate the potential molecular mechanism. Moreover, we analyzed the relationship between our identified signature and immune microenvironment, TMB, and some clinical characteristics. Finally, we detected the expression of the six necroptosis-related lncRNAs in cells and tissues.

**Results:** We constructed a NRlncRNAs signature consisting of six lncRNAs (FRMD6-AS1, LINC01480, FAM83A-AS1, FRMD6-AS1, MED4-AS1, and LINC01415) in LUAD. LUAD patients with high risk scores had lower chance of survival with an AUC of 0.739, 0.709, and 0.733 for 1-year, 3-year, and 5-year respectively. The results based on GO, KEGG, and GSVA enrichment analysis demonstrated that NRlncRNAs signature-related genes were mainly correlated with immune pathways, metabolic-and cell growth-related pathways, cell cycle, and apoptosis. Moreover, the risk score was correlated with the immune status of LUAD patients. Patients with higher risk scores had lower ESTIMATE scores and higher TIDE scores. The risk score was positively correlated with TMB. LINC01415, FRMD6-AS1 and FAM83A-AS1 were significantly overexpressed in lung adenocarcinoma, while the expression levels of MED4-AS1 and LINC01480 were lower in lung adenocarcinoma.

**Conclusion:** Overall, an innovative prognostic signature based on NRlncRNAs was developed for LUAD through comprehensive bioinformatics analysis, which can act as a predictor of immunotherapy and may provide guidance for clinicians.

## Introduction

Lung adenocarcinoma (LUAD) is the most common type of lung cancer nowadays (Siegel et al., 2021). Although there are a variety of comprehensive therapeutic styles such as surgery, chemotherapy, radiotherapy and immunotherapy, some patients cannot be effectively treated and have a low 5-year overall survival rate due to the lack of specific targets (Nasim et al., 2019; Franzi et al., 2022). Therefore, to improve the prognosis and treatment of LUAD patients, it is vital to establish novel, efficient biomarkers and therapeutic approaches.

As a new style of cell death, necroptosis is universally mediated by receptor-interacting protein kinases1/3 of the receptor family (RIPK1/RIPK3) and is mainly governed by the effector protein Mixed Lineage Kinase Domain Like Pseudokinase (MLKL) (Frank and Vince, 2019; Yuan et al., 2019). Furthermore, necroptosis is able to modulate tumor immune responses which may lead to potential immunotherapeutic benefits (Gong et al., 2019; Tang et al., 2020a; Galluzzi and Garg, 2021). In one aspect, during necroptosis, cancer cells release cytokines and chemokines that stimulate inflammatory and tumor-modulating effects in tumor microenvironment. By luring macrophages and dendritic cells, on the other hand, necroptotic tumor cells encourage effector T cells to penetrate tumor tissues, which strengthens the immunosuppression of the tumor. Therefore, targeting necroptosis could lead to novel cancer therapies, especially immunotherapy. Nevertheless, the specific regulatory mechanism of necroptosis in lung cancer remains unclear.

Long non-coding RNAs (lncRNAs) are a class of non-proteincoding RNAs (ncRNAs) whose length is more than 200 nucleotides (Grote and Boon, 2018). A growing body of evidence has demonstrated that lncRNAs play a crucial role in lung cancer progression and the immune pathway (Chen et al., 2017; Bocchetti et al., 2021; Park et al., 2022). LncRNAS can alter cancer cells’ resistance to immune responses, leading to immune evasion. In addition, several studies have demonstrated that lncRNAs can also act as competitive RNAs to influence genes involved in necroptosis (Jiang et al., 2021; Zhao et al., 2021; Chen et al., 2022). However, few studies have been done on necroptosis-related lncRNAs (NRlncRNAs) and tumor immune microenvironment (TIME) in LUAD.

In this study, an innovative prognostic signature based on NRlncRNAs was developed for LUAD. Additionally, we validated its clinical significance, confirming that this signature can act as a predictor of immunotherapy and may provide guidance for clinicians.

## Methods and materials

### Data sources

We downloaded the RNA sequencing data (59 normal tissues and 535 tumor tissues) and corresponding clinical information of LUAD samples from the TCGA database (https:/portal.gdc.cancer.gov/). Necroptosis-related genes were extracted from previous studies (Fan et al., 2014; Frank and Vince, 2019; Gong et al., 2019; Molnár et al., 2019; Yuan et al., 2019; Tang et al., 2020a). The mutation data in MAF format of LUAD samples was also obtained from TCGA. Next, we downloaded the Genome Reference Consortium Human Build 38 (GRCh38) to annotate lncRNAs and mRNAs by preforming Perl scripts.

Identification of Differentially Expressed Necroptosis-related LncRNAs in LUAD.

4191 lncRNAs and 19116 mRNAs were identified from the TCGA-LUAD RNA-seq data. The co-expression correlations between NRGs and lncRNAs in LUAD samples were investigated through Pearson correlation analysis. The cutoffs for this study were |Coefficient| >0.4 and *p*-value <0.001. The “igraph” R package was used to get the co-expression network of NRGs and predictive lncRNAs. Lastly, we analyzed differentially expressed NRlncRNAs using the “limma” package (|logFc = 1|, FDR <0.05).

### Construction and validation the prognostic NRlncRNAs signature

First, the prognosis-related NRlncRNAs were identified in TCGA-LUAD through univariate Cox proportional regression analysis (*p* < 0.05). Significant lncRNAs were visualized in heatmap by using “heatmap” package. The Sankey plot was created by the “limma”, “dplyr,” “ggalluvial,” and “ggplot2” packages to visualize the correlation between NRGs and NRlncRNAs. Then, “caret” package was utilized to allocate all patients into the training and the testing sets. The “glmnt” was performed to select significant NRG-lncRNA into the Least absolute shrinkage and selection operator (LASSO) cox regression. The LASSO Cox regression approach was used to find the best panel of prognostic lncRNAs and create an optimum signature. The standardized expression levels of NRLs and the related regression coefficients produced from the LASSO regression analysis were then used to calculate each LUAD patient’s survival risk score. The formula is given:

Risk score = Σ(Coef (lncRNAi)×Exp (lncRNAi)).

Coef and Exp denote the coefficient and the standardized expression levels of each NRL. The training set’s median risk score was used as the demarcation point to divide LUAD samples into low- or high-risk subgroups. To compare the overall survival (OS) of the high-risk and low-risk subgroups among the training and testing sets, Kaplan-Meier (K-M) curves were generated by performing the “survival” package. A heatmap was utilized to display the significant lncRNA in this model. Time-dependent ROC curves were generated to assess the survival predictive ability of the NRlncRNAs signature. Univariate and multivariate cox regression analysis were performed to detect the independence of this prognostic risk model. We also contrasted the differences among different risk groups and clinical characteristics by using “limma” R package (Ma et al., 2020). To determine if our NRlncRNAs signature risk model is superior to previously reported signatures in LUAD, we compared its predictive power to that of other signatures, including two five-lncRNA signatures (Song et al., 2021; Wang et al., 2022) and a seven-lncRNA signature (Yao et al., 2021). The lncRNAs in these signatures were obtained from the corresponding published literature, and the AUC of 1-, 3-, and 5-year ROC curves, as well as the OS, were calculated for each signature.

### Development of a nomogram score system

Subsequently, a nomogram score system based on age, stage, gender, and risk score of each patient with LUAD was constructed to predict the prognosis of individual patient outcomes. The procedure was run by using “survival” and “rms” packages. The calibration curve was conducted to assess the consistency between the actual outcomes and the predicted prognosis. The AUC of the ROC curve was used to compare the prediction abilities of the nomogram with other prognostic factors. Moreover, decision curve analysis (DCA) was used to evaluate the net clinical utility of the nomogram.

### Function enrichment analysis and gene set variant analysis (GSVA)

To investigate the difference in potential molecular function and cancer-associated signaling, we analyzed the DEGs between high-risk and low-risk groups. Then Gene Oncology (GO) and Kyoto Encyclopedia of Genes and Genomes (KEGG) enrichment analyses were conducted via R packages including clusterProfiler, org. Hs.eg.db and enrichplot. In addition, we performed GSVA to explore the NRlncRNAs signature in KEGG and GO. “c2. cp.kegg.v7.2. symbols.gmt” and “c5. go.v7.4. symbols.gmt” gene sets were downloaded from the MSigDB database. The procedure was conducted by using R packages including GSVA, limma, GSEABase and heatmap. Adjusted *p* < 0.05 was considered as statistical significance.

### Immune microenvironment, immune check-point and immune therapy response analysis

To estimate the connection between the NRlncRNAs signature and immune microenvironment of LUAD samples, a gene expression matrix-based ESTIMATE algorithm was utilized to determine the infiltration levels of stromal cells and immune cells in tumors (Yoshihara et al., 2013). The immune and stromal scores reflected the infiltration levels of immune cells and stromal cells, respectively, while the ESTIMATE score was a stroma-immune composite score. Tumor-infiltrating immune cell dataset was obtained from TIMER2.0 (http://timer.cistrome.org) database. We applied TIMER (Li et al., 2017), CIBERSORT (Chen et al., 2018), QUANTIseq (Plattner et al., 2020), MCP-counter (Dienstmann et al., 2019), xCELL (Aran et al., 2017), and EPIC (Racle et al., 2017) algorithms to compare immune cell abundance between high-risk and low-risk groups based on the NRlncRNAs signature. The expression of immune checkpoint genes between different risk groups was examined to assess the potential effects on immunotherapy. To predict immunotherapy response in patients with malignant tumors, tumor immune dysfunction and exclusion (TIDE) score (http://tide.dfci.harvard.edu/) was calculated.

### Analysis of tumor mutation burden, prediction of the effect of chemotherapy and target therapy

The “maftools” R package was used to analyze the tumor mutation burden (TMB) of LUAD samples (Mayakonda et al., 2018). By comparing TMB between high- and low-risk groups, the top 20 genes with the highest mutation rate and their mutation types were obtained. Then the Kaplan–Meier survival curves were used to assess the effect of TMB on the OS of LUAD patients. The “pRRophetic” R package was performed to predict the IC50 of commonly used chemotherapeutic drugs (Geeleher et al., 2014). Wilcoxon signed-rank test was used to determine the difference between the groups.

### Consensus clustering for NRlncRNAs signature

Unsupervised consensus clustering was conducted on 490 LUAD patients using “ConsensusClusterPlus” based on the expression of the NRlncRNAs signature to find potential molecular subgroups (Wilkerson and Hayes, 2010). The “survival” and “survminer” packages were used to perform Kaplan–Meier survival analysis between distinct clusters in R software. Principle component analysis was performed to explore the discrimination among different clusters and risk groups. A Sankey diagram was plotted to display the molecular subtypes and survival status of patients in different risk groups. The “heatmap” R package was used to examine the differences in molecular subtypes for diverse clinicopathological characteristics. GSVA analysis was performed to identify the potential KEGG pathways associated with different clusters. TMB value, the abundance of infiltrating immune cells, the expression of checkpoints and drug sensitivity analysis of different clusters were evaluated as mentioned earlier.

### Cell culture

Immortalized lung epithelial cells (BEAS-2B) and lung adenocarcinoma cells (A549/PC9) were all obtained from American Type Culture Collection (ATCC). BEAS-2B and A549 cells were incubated in DMEM high glucose medium with 10% fresh fetal bovine serum. PC9 cells were incubated in RPMI 1640 medium with 10% fresh fetal bovine serum. All the cells were cultured in a constant-temperature incubator (37°C, 5% CO2) for proper time to get the total RNA.

### Quantitative real-time PCR (RT-qPCR)

Total RNA of cells (BEAS-2B, A549 and PC9) and tissues was extracted by using an RNA extraction kit (Tiangen) following the protocol. 1000ng of total RNA was reversely transcribed and then PCR amplification of obtained cDNA was processed by using the kit (Cowin Bio.) and right primers. Sequences of primers we used in this study were designed by Primer-BLAST and were listed in supplemental table (Supplementary Table S1).

### Statistical analysis

R 4.0.4 (https://www.r-project.org/) was used for all statistical analyses. The chi-square test or Fisher’s exact test were used to test categorical variables. On continuous variables, the t-test or Wilcoxon test was used. Statistical significance was defined as *p* < 0.05.

## Results

### Identification of differentially expressed necroptosis-related LncRNAs in patients with LUAD

**FIGURE 1:**
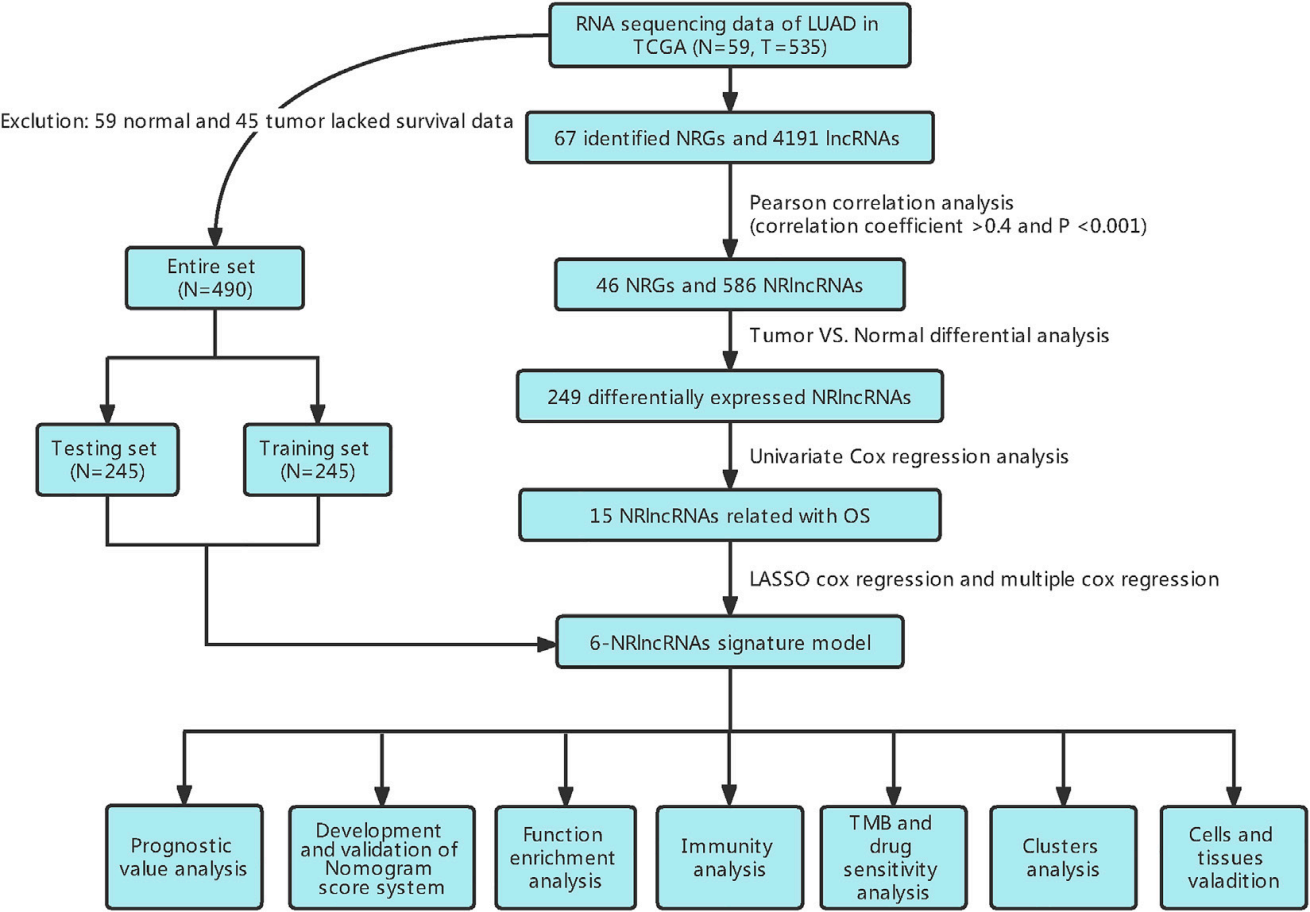
Flow diagram of the study design.

**FIGURE 2:**
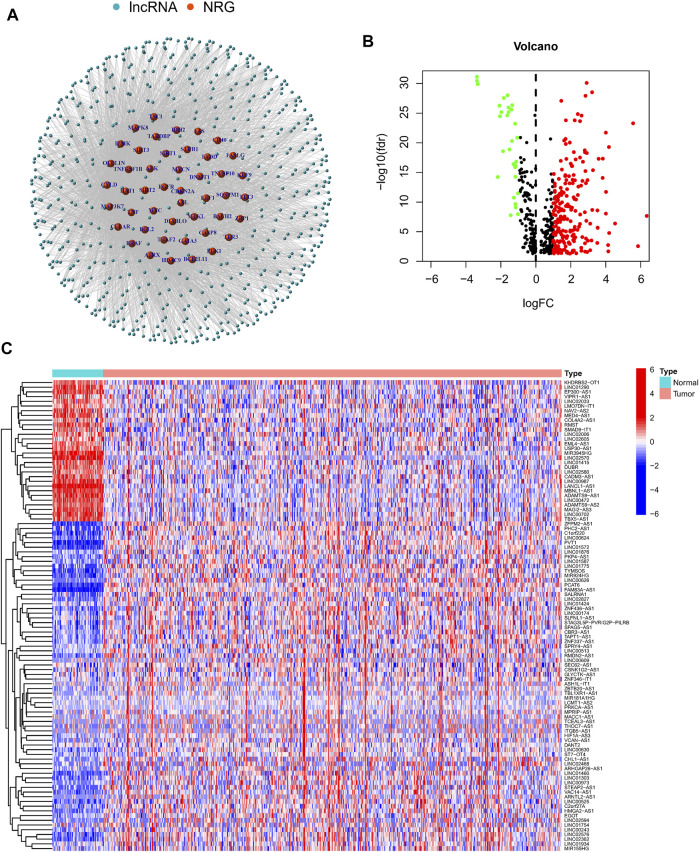
Identification of differentially expressed necroptosisNRlncRNAs in Lung adenocarcinoma. **(A)** A co-expression network of the 586 NRlncRNAs-mRNA was constructed and visualized by using “igraph” R package. **(B,C)**. The volcano plot and heatmap showed the differentially expressed NRlncRNAs in tumor and normal tissues.

In this study, 490 LUAD samples with comprehensive clinical data were included for further analysis. A flowchart of the study is presented in Figure 1. Firstly, we identified 67 NRGs from previous studies (Supplementary Table S2). Then, we analyzed the expression matrix of NRGs and lncRNAs by performing Pearson correlation analysis and obtained 586 NRlncRNAs (Supplementary Table S3). The network of mRNA-lncRNA co-expression showed a potential connection of 46 NRGs and 586 NRlncRNAs (Figure 2A). Among the 586 NRlncRNAs, 249 were found to be differentially expressed using the criteria: |logFc = 1|, FDR < 0.05 (Figure 2B). The heatmap displayed the 249 NRG-lncRNAs expression landscape between tumor and normal tissues (Figure 2C).

### Development and validation of the prognostic risk model

**FIGURE 3:**
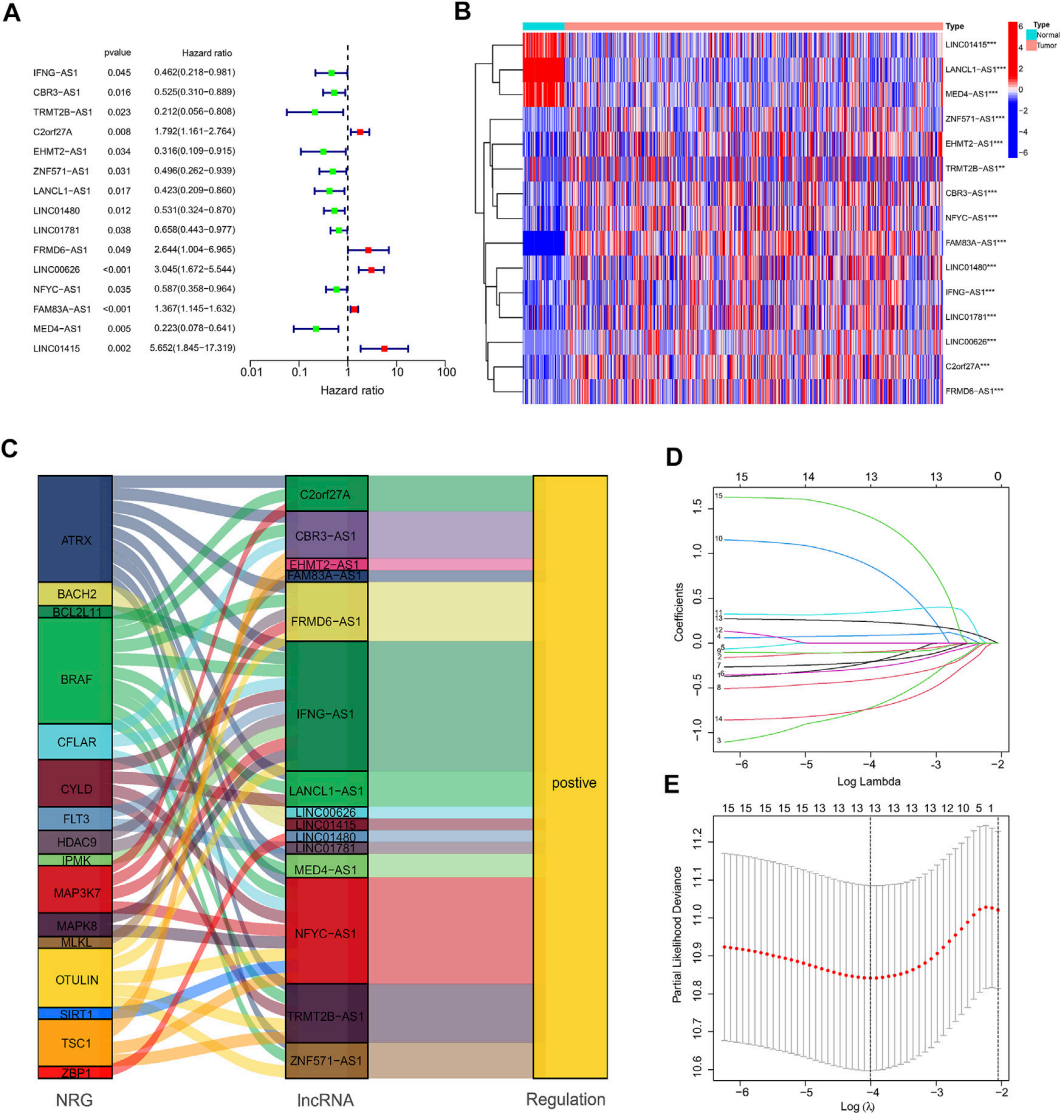
Construction of the NRlncRNAs signature in the TCGA. **(A,B)**. The forest plot and heatmap showed 15 NRlncRNAs with prognostic value in LUAD. **(C)**. The Sankey diagram of 16 NRGs and 15 NRlncRNAs. **(D,E)**. The LASSO coefficient and the 10-fold cross-validation for variable selection in the LASSO model.

**FIGURE 4:**
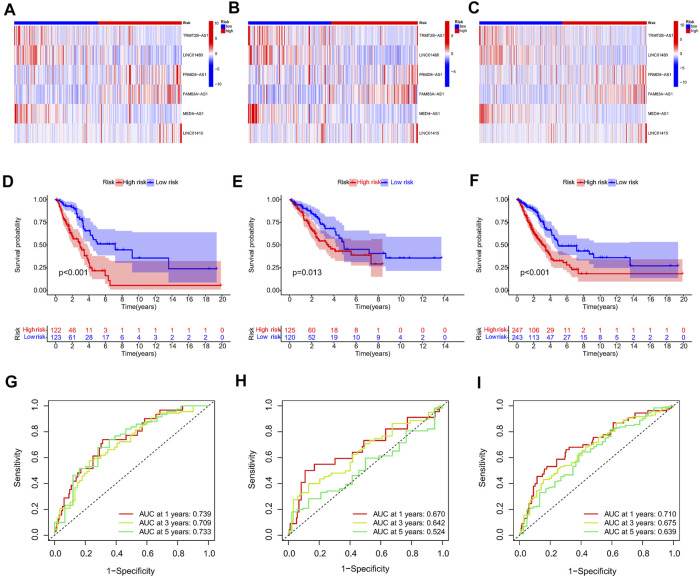
Prognosis value of the 6 NRlncRNAs model in the train, test, and entire sets. **(A–C)** The heatmap of six NRlncRNAs between two groups in the train, test, and entire set, respectively. **(D–F)** Survival status and time of patients between two groups in the train, test, and entire set, respectively, **(G–I)** The time-dependent ROC curve of patients between two groups in the train, test, and entire set, respectively.

**FIGURE 5:**
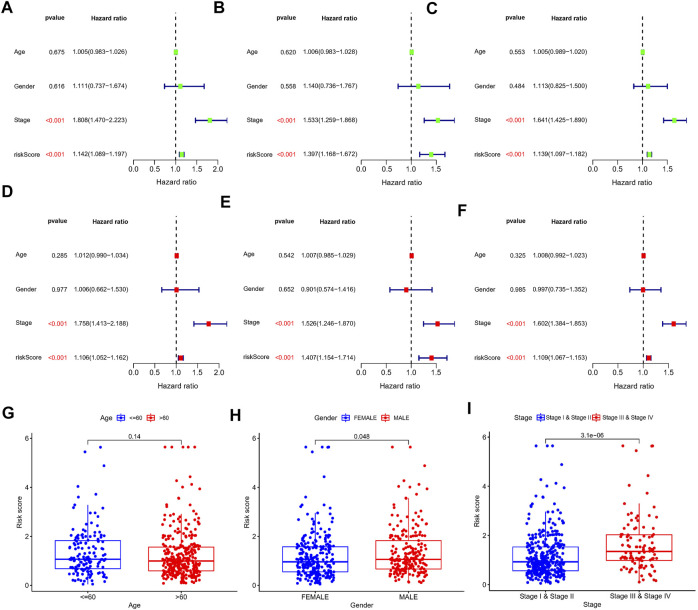
Univariate and multivariate Cox regression analysis in the train, test, and entire set, respectively. **(A–C)** Univariate Cox regression analysis in the train, test, and entire set, respectively. **(D–F)** Multivariate Cox regression analysis in the train, test, and entire set, respectively. **(G–I)** Distribution of risk score stratified by age, gender and clinical stage in entire set.

First, a total of 15 NRlncRNAs were identified as the prognostic lncRNAs for patients with LUAD by univariate Cox regression analysis. (Figure 3A). The expression heatmap of 15 prognostic NRlncRNAs was presented in Figure 3B. The Sankey diagram showed the positive regulatory relationship between 16 NRGs and 15 NRlncRNAs (Figure 3C). Next, we randomly divided 490 LUAD samples into a training dataset (n = 245) and a testing dataset (n = 245), and we performed LASSO cox regression to select 13 NRlncRNAs for multiple cox regression (Figures 3D,E; Supplementary Table S4). Finally, the risk model was updated using multivariate Cox regression analysis and included six NRlncRNAs. Risk score = TRMT2B-AS1 * (-1.5285) + LINC01480 * (-0.7024) + FRMD6-AS1*1.3853 + FAM83A-AS1 * 0.3043 + MED4-AS1 * (-1.2861) + LINC01415 * 2.0938. The expression of six NRlncRNAs between high- and low-risk groups was presented in Supplementary Figure S1. Based on the median risk score of the training set, we divided samples into high-risk group and low-risk group in the training, testing and entire datasets (Supplementary Figure S2). And we discovered that the majority of the dead patients belonged to the high-risk category (Supplementary Figure S2). The expression of six NRlncRNAs in high- and low-risk groups was shown in Figures 4A–C. Additionally, the K-M curves indicated that patients with a high-risk score had a lower chance of survival than patients with a low-risk score. (Figures 4D–F). NRlncRNAs To further investigate the prognosis value of this signature, we performed the time-dependent receiver operating characteristic (ROC) analysis. The area under curve (AUC) values for the 1-year, 2-year, and 3-year survival rates showed good specificity and sensitivity of this signature in predicting OS either in the training, testing, or entire group (Figures 4G–I). Next, we investigated whether the signature was independent of other clinical characteristics using univariate and multivariate cox regression analysis. The results indicated that the risk score of our signature was correlated with the OS and it acted as an independent prognostic predictor for patients with LUAD (Figures 5A–F). To explore the differences in risk score among different subgroups of patients, we discovered that male and advanced patients had a higher risk score than female and early-stage patients in the entire TCGA cohort (Figures 5G–I). Furthmore, the survival rate was significantly lower in the high-risk group than in the low-risk group in the age ≥ 60 (*p* < 0.001), age < 60 (*p* = 0.033), male (*p* < 0.001), female (*p* < 0.001), stage I and stage II (*p* = 0.001), and stage III and stage IV (*p* = 0.002) subgroups of patients, according to stratified survival analysis in combination with clinical characteristics (Supplementary Figure S3). Additionally, compared with other previously identified signatures, our risk model based on the 6 lncRNAs signature had better predictive power (Supplementary Figure S4).

### The signature-based nomogram score system for predicting the prognosis of LUAD patients

**FIGURE 6:**
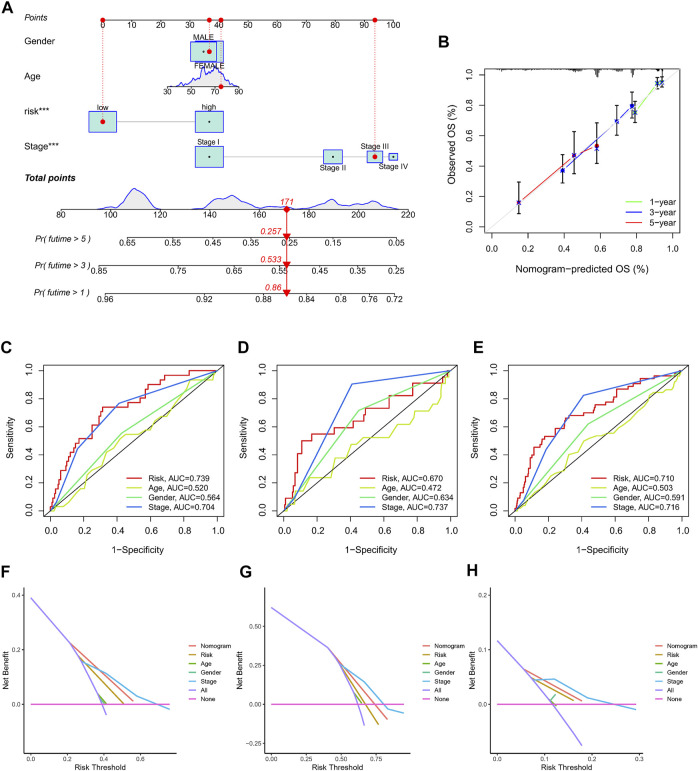
The nomogram used to predict the OS prognosis of LUAD patients at 1, 3, and 5 years. **(A)** A nomogram based on characteristics and clinical information. **(B)**. Calibration curve of nomogram. **(C–E)** ROC analysis of risk score, nomogram, age, gender and clinical stage predicting OS at 1, 3, and 5 years. **(F–H)** The DCA of NRlncRNAs prognostic risk scores and several clinicopathological factors such as gender, age, and stage.

In order to create a more reliable model for prognosis prediction, a nomogram comprised of the identified NRlncRNAs signature and several clinical characteristics was found to be effective for predicting 1-, 3-, and 5-year survival probabilities in the entire dataset (Figure 6A). Furthermore, the calibration curve revealed an accordant agreement between observation and prediction for 1-, 3-, and 5-year OS in LUAD (Figure 6B). In addition, the nomogram score system had superior predictive power (1-year AUC = 0.735, 3-year AUC = 0.724, and 5-year AUC = 0.697) than the risk score, age, gender and stage (Figures 6C–E). The DCA curves suggested that the nomogram and risk score had good consistency in forecasting survival rate at 1-, 3-, and 5-year (Figures 6F–H).

### Function enrichment analysis

**FIGURE 7:**
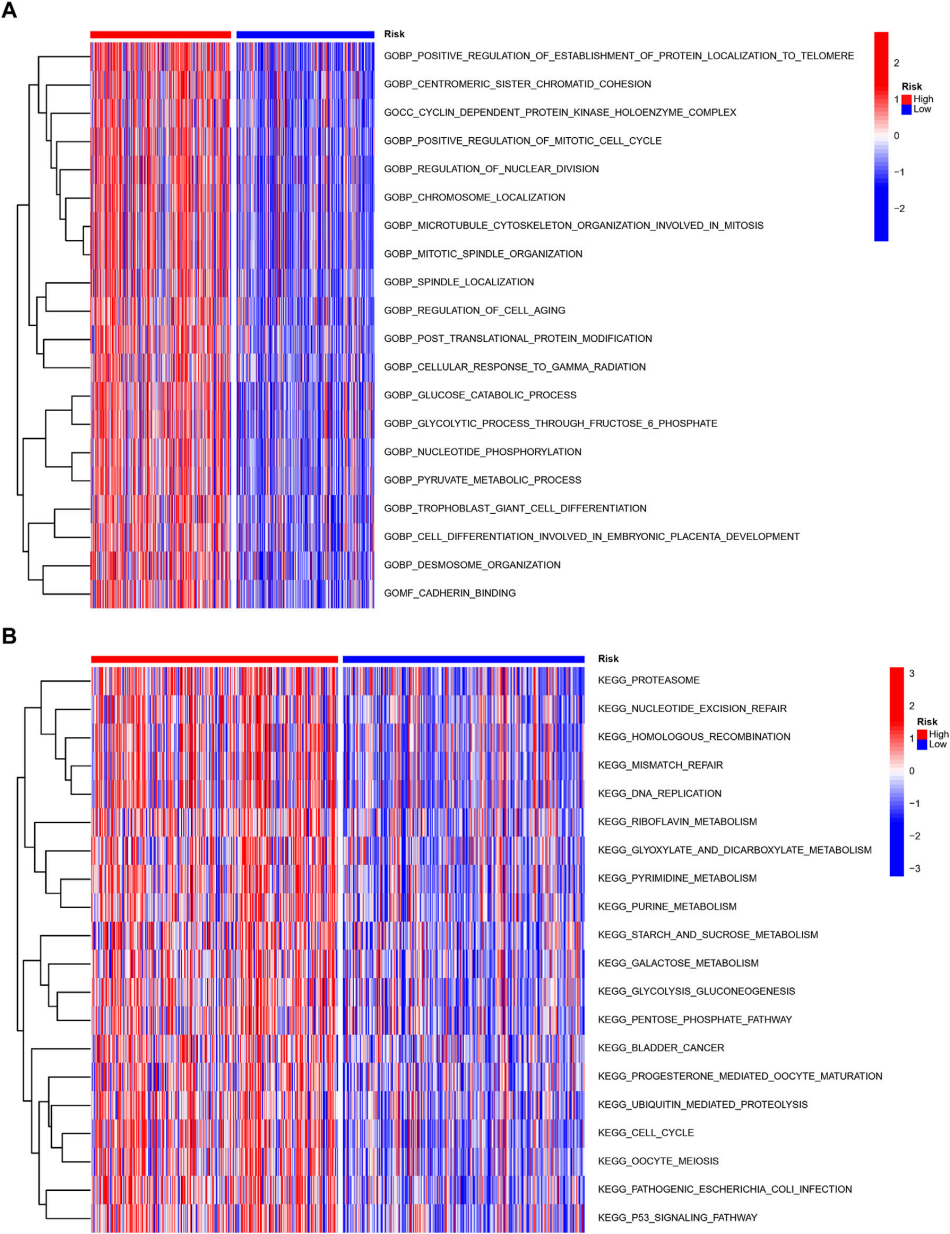
Molecular characteristics of patients in the high- and low-risk groups. The GSVA analysis in the high- and low-risk group to enrich GO characteristic gene sets **(A)** and KEGG gene sets **(B)**.

We performed GO and KEGG to investigate the probable mechanisms of the NRlncRNAs signature-related genes’ expression. The results revealed that these genes were associated with immune pathways, such as MHC class II protein complex binding, Th17 cell or Th1 and Th2 cell differentiation, and antigen processing and presentation. Moreover, cancer-associated biological functions such as cell cycle, apoptosis, and glycolysis were found to be significant in LUAD patients (Supplementary Figure S4). We then run a GSVA enrichment analysis to delve deeper into the underlying differences in biological characteristics that underlie the different risk groups. As shown in Figures 7A,B, the high-risk group had a considerable enrichment of metabolic- and necroptosis-related pathways, such as pyruvate metabolism, glucose metabolism, DNA replication, cell cycle and P53 signal pathway.

### Tumor microenvironment, immune cell infiltration, and immunotherapy response of LUAD patients

**FIGURE 8:**
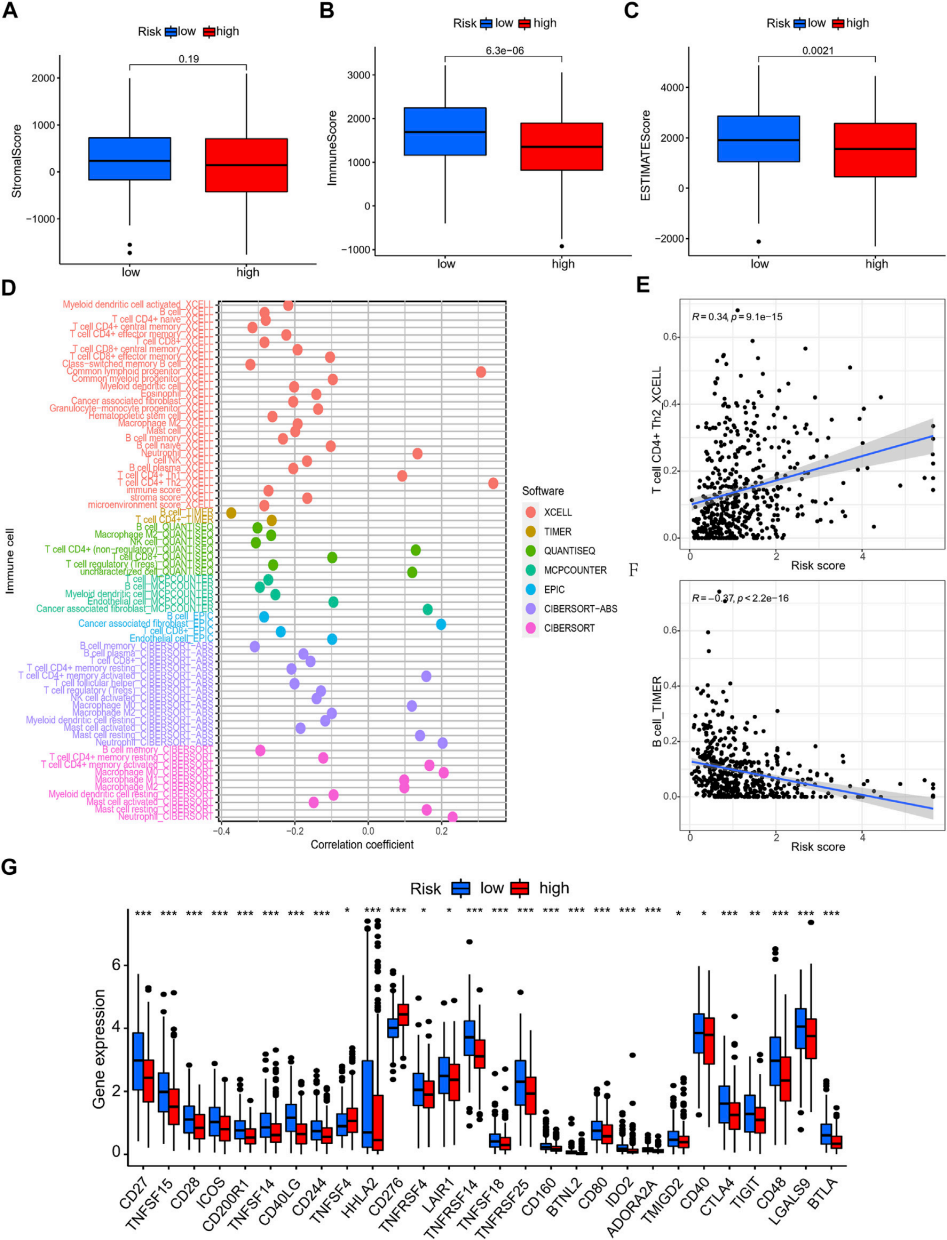
The immune landscape analysis of NRlncRNAs signature in LUAD patients. **(A–C)** The TME score (immune score, stromal score, and ESTIMATE score) between different risk group. **(D–F)** Relationship between the NRlncRNAs risk score and immune cells infiltration. **(G)** The differential expression of immune checkpoint between high- and low-risk group.

As above, we discovered that the risk score was associated with immune-associated pathways. Next, we compared the difference in immunological state among different risk categories. The results based on ESTIMATE algorithm revealed that the immune and ESTIMATE scores were lower in the high-risk group than in the low-risk group (Figures 8A–C). The correlation examination of immune cell infiltration in two risk groups indicated that CD4^+^ T cells, NK cells, and B cells were negatively regulated in multiple algorithms, whereas neutrophil cells were positively regulated (Figure 8D). The CD4^+^ Th2 cells and B cells had the most obvious coefficient connection with the risk score (Figures 8E,F; Supplementary Table S5). Then, to investigate the difference in immunological status between the high-risk and low-risk groups, we examined their TIDE scores, exclusion scores, and dysfunction scores. According to the findings, the TIDE and exclusion score were higher in the high-risk group than in the low-risk group (Supplementary Figure S6). Low-risk patients had a greater dysfunction score than high-risk patients, whereas the exclusion score had the opposite pattern (Supplementary Figure S6). These data indicated that the high-risk group had higher immune escape risk and poor prognosis. The different expression of 28 immune check points between the two risk groups showed that the expression of TNFSF4, CD276 was higher in the high-risk group, while the other multiple checkpoints presented higher expression in the low-risk group (Figure 8G).

### Analysis of tumor burden mutation and drug sensitivity

**FIGURE 9:**
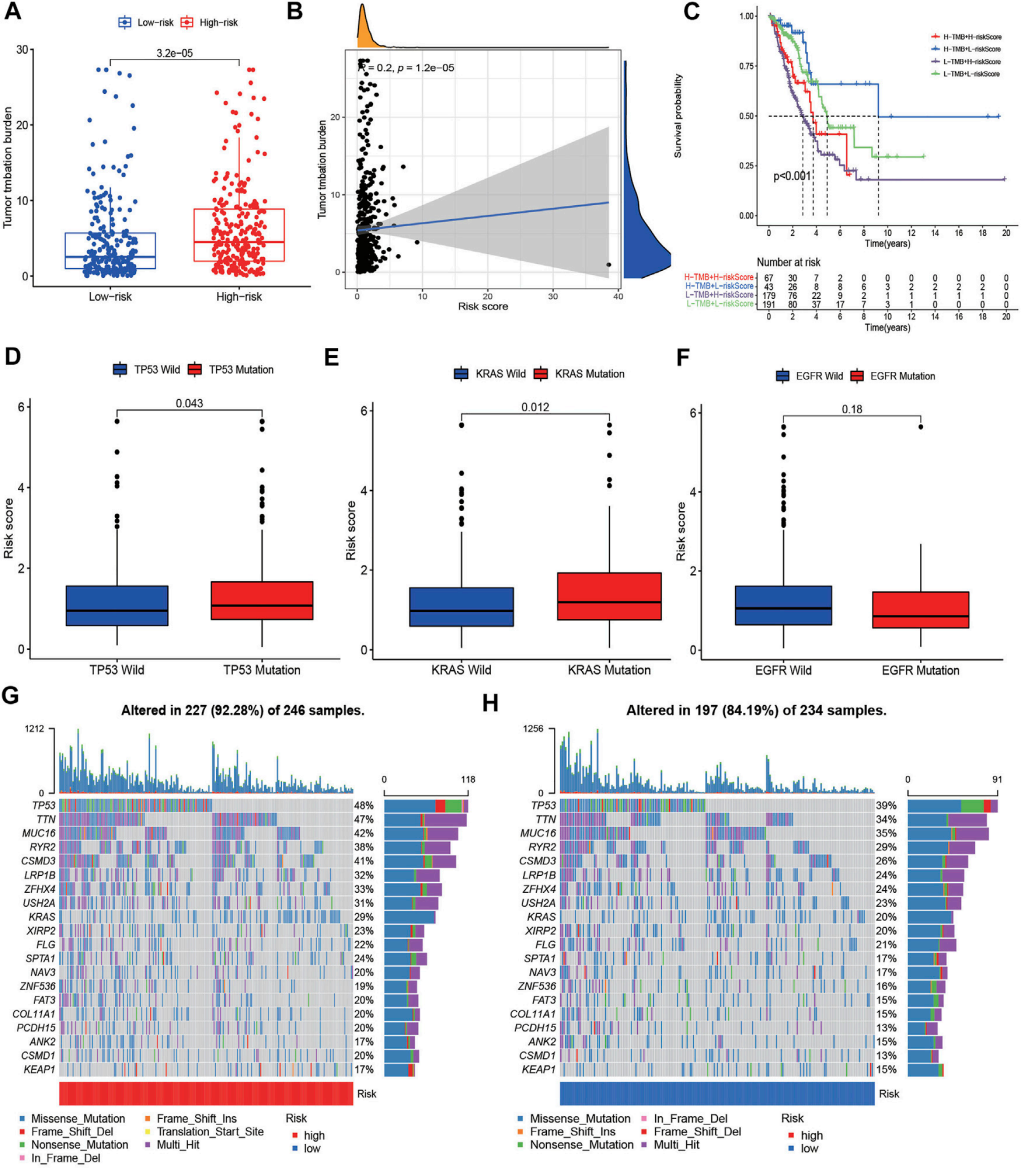
The TMB level and Somatic mutation analysis of risk score. **(A)** Differences of tumor mutation burden (TMB) in the high-risk and low-risk groups. **(B)** Relationship between TMB and risk score. **(C)** Survival analysis between patients with high- and low-TMB. **(C)** Two-factor survival analyses of risk score and TMB levels. **(D–F)** Differences of risk score between different status of TP53, KRAS, and EGFR. **(G,H)** Landscape of top 20 mutated gene mutation profiles between high- and low-risk group.

Previous studies have reported that TMB plays a crucial role in immunotherapy, chemotherapy, and target therapy responses. Hence, we further investigated the correlation between risk score and TMB. It showed that the high-risk group had higher TMB than low-risk group, and the risk score was positively correlated with TMB (Figures 9A,B). Patients with lower TMB and higher risk score had worse prognosis (Figure 9C). According to the status of TP53, KRAS and EGFR, the patients with TP53 and KRAS mutations had higher risk scores, while patients with EGFR mutation showed lower risk scores (Figures 9D–F). The waterfall plot presented the top 20 genes with the most mutations in different risk groups. The high-risk group presented higher genetic alteration rate than the low-risk group (92.28 vs. 84.19%), and TP53 and TTN were the top two genes in each risk group (Figures 9G,H). Drug sensitivity analysis between different risk groups showed that patients with high risk scores were more sensitive to docetaxel, doxorubicin, erlotinib and gefitinib et al. (Supplementary Figure S7).

### Characterization of clusters in different risk groups

**FIGURE 10:**
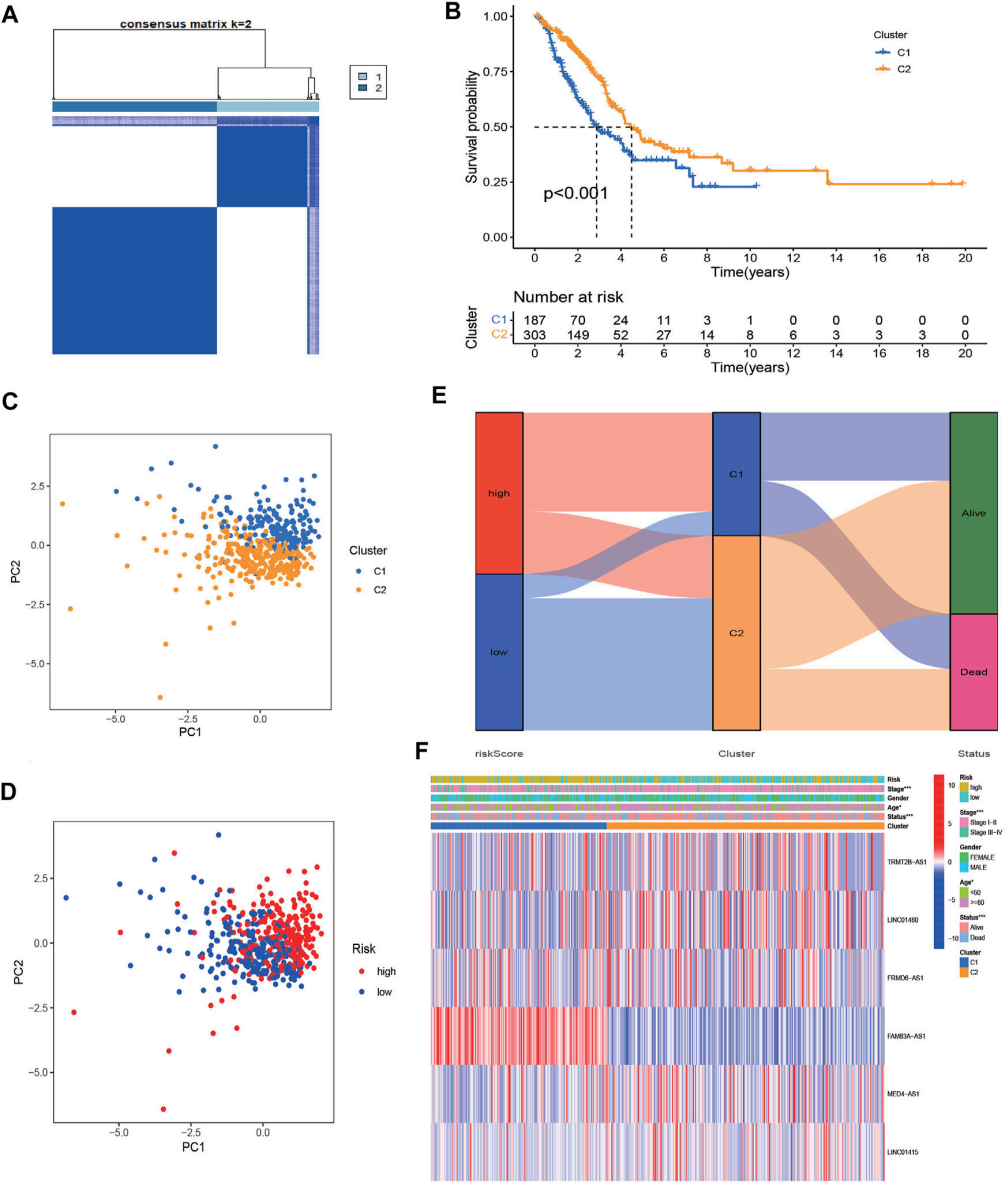
Identification of two clusters based on prognostic NRlncRNAs signature. **(A)** Consensus clustering (K-means) algorithm was performed for overall patients. Consensus matrix plots. K = 2 was determined as optimal clustering number. **(B)** K–M survival analysis in clusters C1 and C2. **(C,D)** The distribution of different patients from two clusters and risk groups. **(E)**. The Sankey diagram showed distribution of patients in different risk groups, two clusters and survival status. **(F)**. Heatmap of the lncRNA expression of prognostic NRlncRNAs signature between two clusters.

To further assess the distinct molecular patterns based on the expression of the NRlncRNAs signature, we performed unsupervised consensus clustering to divided patients with different risk scores into two clusters, with k = 2 shown to be the best for clustering stability (Figure 10A). As presented in Figure 10B, the patients in cluster 1 had a poorer prognosis than those in cluster 2. Furthermore, the results of PCA suggested that clusters and risk groups can be completely distinguished (Figures 10C,D). The Sankey plot revealed the distribution of patients in different risk groups, clusters and survival status, with cluster 1 accounting for the majority of high-risk patients (Figure 10E). Furthermore, the heatmap presented the expression landscape of six NRG-LncRNAs in two clusters, which indicated that two clusters had significant differences in clinical stage, age, and survival status (Figure 10F). The GSVA results revealed that cluster1 was associated with cell cycle, mismatch repair and some cancer-associated pathways (Supplementary Figure S8). Similar to the risk model, cluster 2 had a higher immune score and ESTIMATE score (Supplementary Figure S9). The status of multiple immune cells infiltration in two clusters exhibited significant differences. The heatmap of the immune infiltration landscape revealed that cluster 1 had a positive correlation with B cells, CD4^+^ T cells, macrophages cells, mast cells activated and cancer-associated fibroblasts, while it had a negative correlation with monocytes (Supplementary Figure S9). The expression level of these immune checkpoints, such as TNFSF4, CD274, CD276, NRP1, TNFRSF18 was higher in cluster 1, while the expression levels of CD44, IDO2, HHLA2 et al. were higher in cluster 2 (Supplementary Figure S9). The drug prediction analysis showed similar results to the risk model. As shown in Supplementary Figure S10, cluster 1 had a lower IC50 of gefitinib, erlotinib, docetaxel and paclitaxel. These results revealed that individuals in cluster 1 were more susceptible to chemotherapy and target therapy.

### The expression level of six prognostic LncRNAs

**FIGURE 11:**
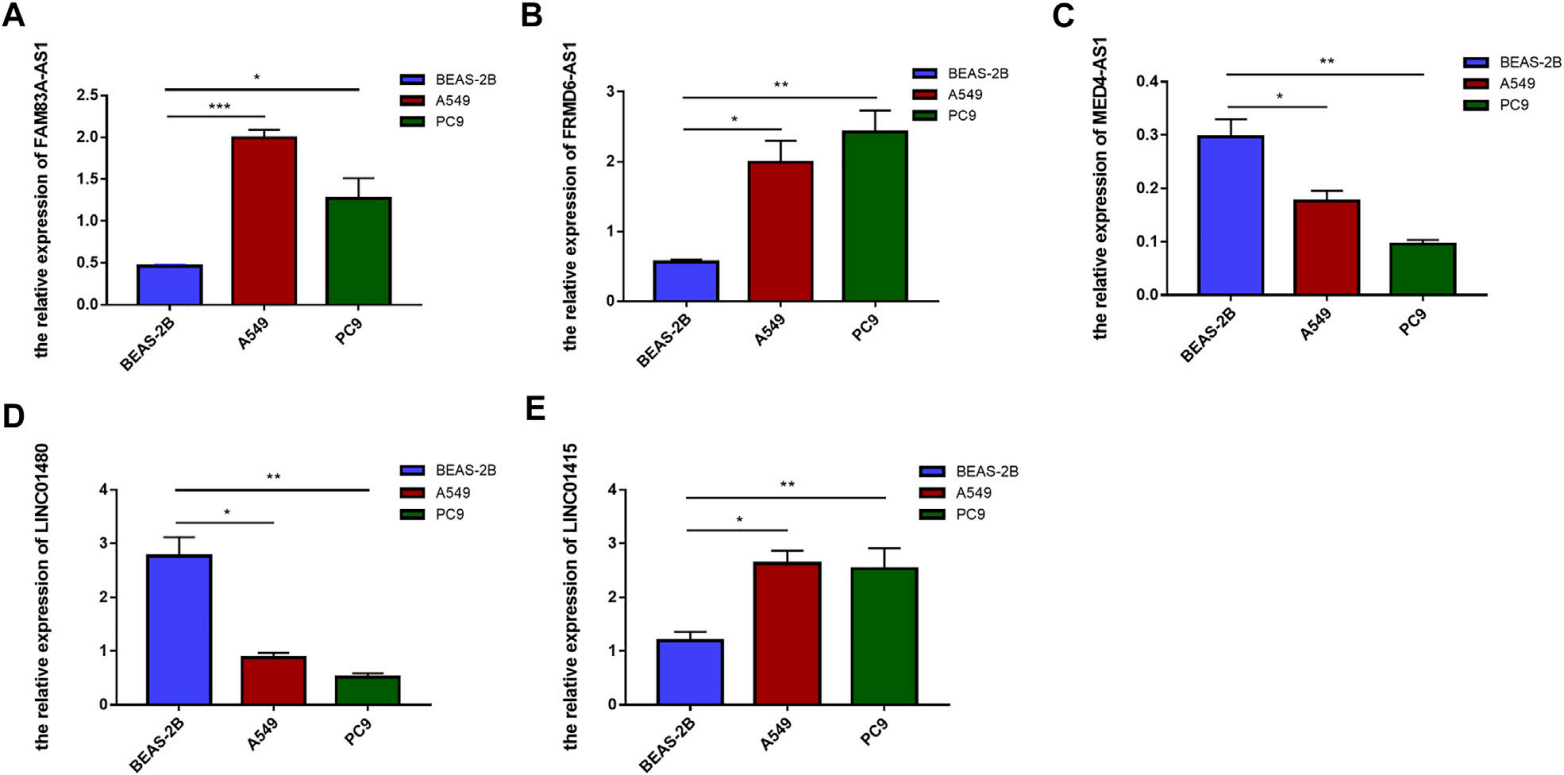
The expression level of six NRlncRNAs in BEAS-2B, A549, and PC9 cell lines. Expression of FAM83A-AS1 **(A)**, FRMD6-AS1 **(B)**, MED4-AS1 **(C)**, LINC01480 **(D)**, and LINC01415 **(E)** in normal lung epithelial cells (BEAS-2B) and lung adenocarcinoma cells (A549/PC9) detected by RT-qPCR. *p < 0.05, **p < 0.01, ***p < 0.001.

**FIGURE 12:**
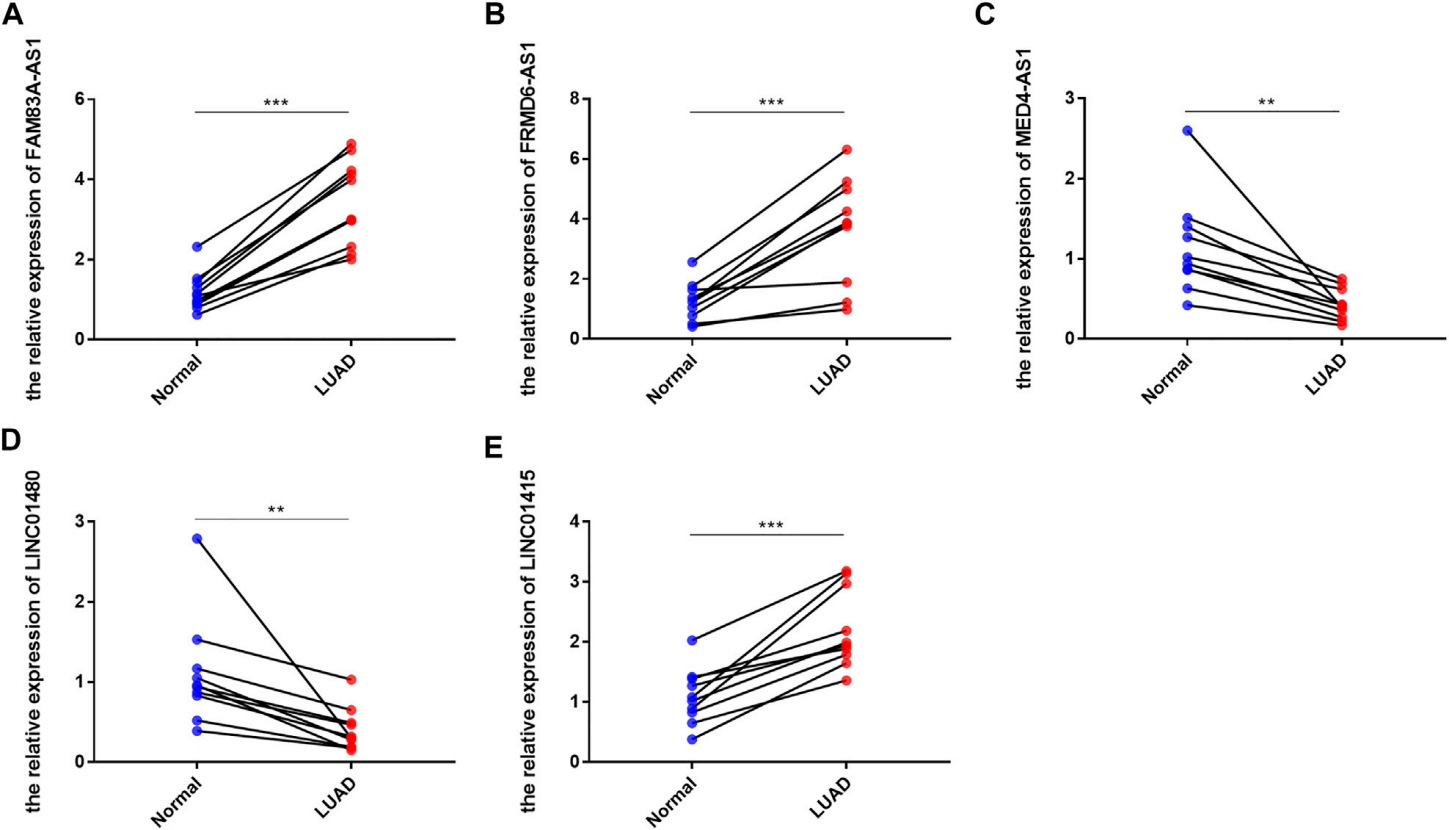
The expression level of six NRlncRNAs in lung adenocarcinoma tissues and adjacent normal lung tissues. Expression of FAM83A-AS1 **(A)**, FRMD6-AS1 **(B)**, MED4-AS1 **(C)**, LINC01480 **(D)**, and LINC01415 **(E)** in lung adenocarcinoma tissues and adjacent normal lung tissues detected by RT-qPCR. *p < 0.05, **p < 0.01, ***p < 0.001.

To further verify the expression of these screened lncRNAs in lung adenocarcinoma cells, total RNA of BEAS-2B, A549, and PC9 were extracted and real-time quantitative PCR was conducted. Surprisingly, our results were consistent with the database. Compared with normal lung-epithelial cells (BEAS-2B), the expression level of LINC01415, FRMD6-AS1 and FAM83A-AS1 was significantly higher in lung adenocarcinoma cells (A549/PC9), while the expression level of MED4-AS1 and LINC01480 was lower in lung adenocarcinoma cells (Figure 11). We also detected the expression of our signature in lung adenocarcinoma tissues and adjacent normal lung tissues, and we obtained consistent results with our observations in cells (Figure 12).

## Discussion

Lung adenocarcinoma remains a serious health problem worldwide with its high mortality and morbidity rates (Mao et al., 2016). In order to improve prognosis for LUAD patients, it is vital to identify a precise and reliable prognostic signature. Recent studies indicated that tumor cells resistant to apoptosis may be sensitive to the necroptosis (Su et al., 2016; He et al., 2017), suggesting that necroptosis may be a potential therapeutic target for lung adenocarcinoma. Besides, lncRNAs also play important roles in tumor genesis and metastasis. Therefore, a necroptosis-related lncRNAs signature was constructed here for the prognosis and treatment of LUAD patients.

In this study, we first identified 15 NRlncRNAs correlated with the OS of patients with LUAD by performing Pearson correlation analysis and univariate Cox proportional regression analysis. Subsequently, a signature including 6 NRlncRNAs (TRMT2B-AS1, LINC01480, FRMD6-AS1, FAM83A-AS1, MED4-AS1, LINC01415) was developed by LASSO analyses and multiple cox regression. Furthermore, LUAD patients were classified into high- and low-risk groups according to their risk scores based on the signature. We found that patients with high risk scores have lower probabilities of survival than those with low risk scores. The ROC analysis showed that our signature had significant prognostic value both in training and testing sets and it showed superiority over previous lncRNA signatures in predicting survival of LUAD patients. Besides, the nomogram system based on our signature and some clinical characteristics also showed steady predictive performance. To sum up, our results above indicated that our NRlncRNAs signature is a good prognostic predictor for LUAD patients.

A mount of studies illustrated the vital role of lncRNAs in tumor progression, but the relationship between the 6 lncRNAs and LUAD is still unclear to us. Among these 6 NRlncRNAs included in our signature, FAM83A-AS1 was found to promote the development of LUAD and suggesting a novel therapeutic approach for LUAD by sponging miR-141-3p (Huang et al., 2022). MED4-AS1 was associated with the prognostic of lung adenocarcinoma (Tang et al., 2020b). LINC01415 was associated with a poor prognosis in ESCC (Tang et al., 2020c). However, the other lncRNAs have not been reported in tumors, which may give us insight into the mechanism of development of lung adenocarcinoma. Therefore, we performed function enrichment analysis based on the differentially expressed genes between different risk groups to investigate the potential mechanism. GSVA showed that metabolism- and cell growth-associated pathways including pyruvate metabolism, glucose metabolism, mismatch repair, and cell cycle was observed in the high-risk group. Nowadays, more and more studies have found that metabolic proteome is involved in tumor development and immune response, which is likely to be a new target for future tumor therapy (Malireddi et al., 2020; Liu et al., 2021). In breast cancer cells glucose deprivation triggers ZBP1-depedent necroptosis (Yu et al., 2021). In colorectal cancer cells, by scavenging free radicals in the mitochondria, glucose confers resistance to 5-FU-induced necroptosis (Zhu et al., 2021). In summary, the necroptosis-related signature may be involved in glucose metabolism in patients with LUAD, thereby influence the necroptosis of lung adenocarcinoma cells, which may be an important direction for future research in lung adenocarcinoma treatment.

Previously, a large number of studies have reported that necroptosis is related to tumor development and immune response (Malireddi et al., 2020; Liu et al., 2021; Yan et al., 2022). In our study, the GO and KEGG analysis indicated that the NRlncRNAs signature-related genes were associated with immune pathways, especially MHC class II protein complex binding. Previous reports indicated that MHC class II protein may be involved in the polarization of tumor-associated macrophage (TAM) to M1 phenotype, which could inhibit the lung cancer cells proliferation and promote the apoptosis (Yu et al., 2021). In addition, comparing immune status among different risk groups, we found that the high-risk group had a higher risk of immune escape and a poorer prognosis. CD4^+^ T cell, NK cell, and B cells were mainly active among the high-risk groups, some of which were closely linked to necroptosis. As studied previously, Rac-1 related-necrotic cells could enhance proinflammatory NK cell killing (Zhu et al., 2021). The inhibition of RIP3 can increase the proportion of CD4^+^ T cells and inhibit the secretion of inflammatory cytokines (IFN-γ, IL-16 and IL-17) (Duan et al., 2022). These results indicated that our NRlncRNAs signature was significantly related with tumor immune microenvironment and it can predict the immune landscape of patients with LUAD.

Moreover, our findings demonstrated that patients in high-risk group had higher TMB, and the risk score was positively correlated with TMB. Besides, patients with lower TMB and high-risk had worse prognosis. TMB is considered a potential biomarker for discriminating NSCLC patients who might benefit more from immunotherapy (Pan et al., 2022). This also suggests that patients with high risk may be more sensitive to immunotherapy. In addition, our results showed that patients in high-risk group were more sensitive to docetaxel, doxorubicin, erlotinib and gefitinib. These results allowed us to select these populations that are more sensitive to drugs and receive better treatment.

Finally, we used RT-qPCR to detect he expression of these signature NRlncRNAs in lung adenocarcinoma cells and tissues. Surprisingly, the results confirmed that the expression levels of these NRlncRNAs were abnormal in LUAD, implying that these lncRNAs may play distinct roles in LUAD. Although our findings shed light on the mechanisms of necroptosis in LUAD, the limitations and flaws still exist. First, although we conducted some experiments to validate it, additional clinical evidence is required to confirm the findings. Second, the underlying mechanism of NRlncRNAs in LUAD is still unknown, and more research is needed in the future.

## Data Availability

The original contributions presented in the study are included in the article/Supplementary Material, further inquiries can be directed to the corresponding authors.

## Abbreviations

LUAD, lung adenocarcinoma; RIPK1/RIPK3, receptor-interacting protein kinases1/3; MLKL, mixed lineage kinase domain like pseudokinase; lncRNAs, long non-coding RNAs; ncRNAs non-proteincoding RNAs; NRGs, necroptosis-related genes; NRlncRNAs, necroptosis-related lncRNAs; TIME, tumor immune microenvironment; TCGA, The Cancer Genome Atlas; GRCh38, the genome reference consortium human build 38; LASSO, the least absolute shrinkage and selection operator; OS, overall survival; K-M curves, Kaplan-Meier curves; DCA, decision curve analysis; ROC curve, receiver operating characteristic curve; AUC, area under curve; GSVA, gene set variant analysis; GO, Gene Oncology; KEGG, Kyoto Encyclopedia of Genes and Genomes; TMB, tumor mutation burden; ATCC, American Type Culture Collection; RT-qPCR, quantitative real-time PCR.
