# Supplementary material for: Cross-talk between necroptosis-related lncRNAs to construct a novel signature and predict the immune landscape of lung adenocarcinoma patients
Source: Front Genet. 2022 Sep 15;13:966896. doi: 10.3389/fgene.2022.966896 (PMC9519990; doi:10.3389/fgene.2022.966896)
Supplement: Supplementary file 1 [file Table2.DOCX]

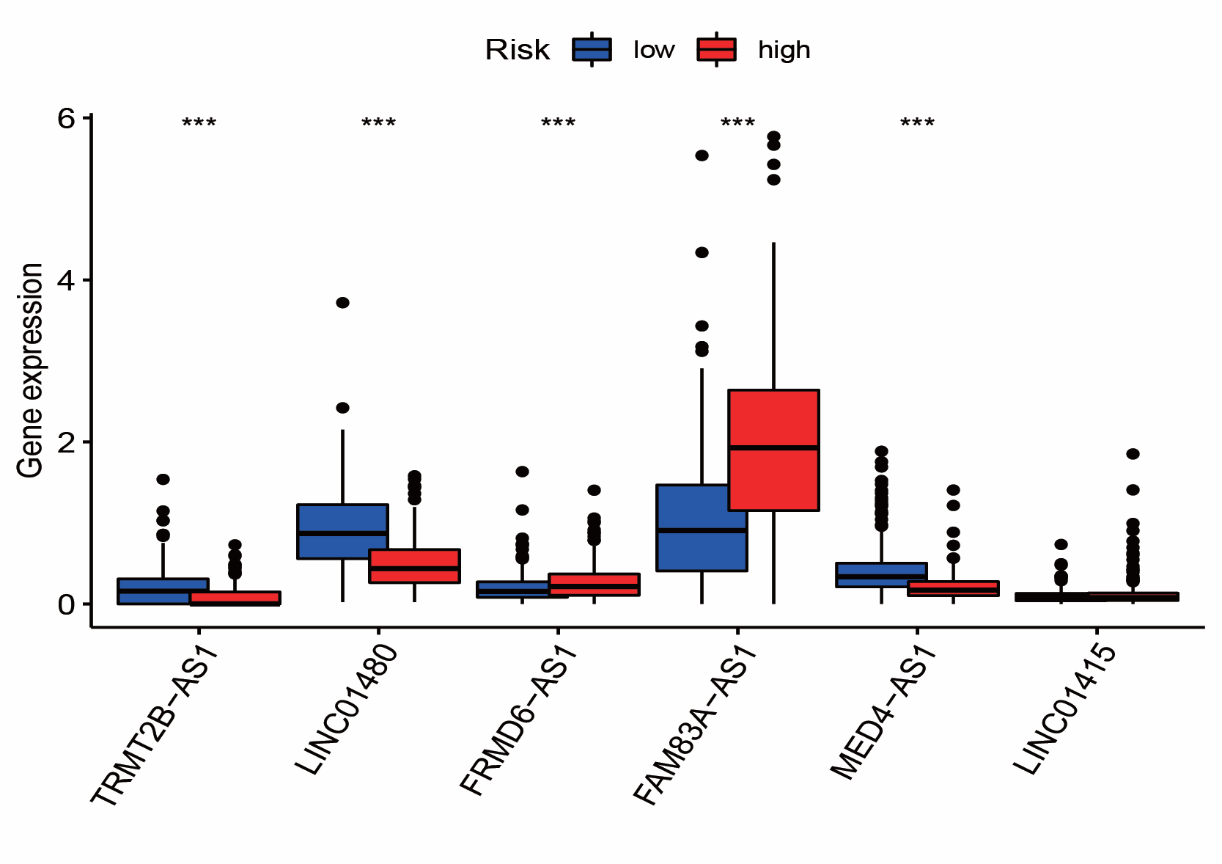


**Figure S1. The expression level of six NRGs-lncRNA in different risk group.**


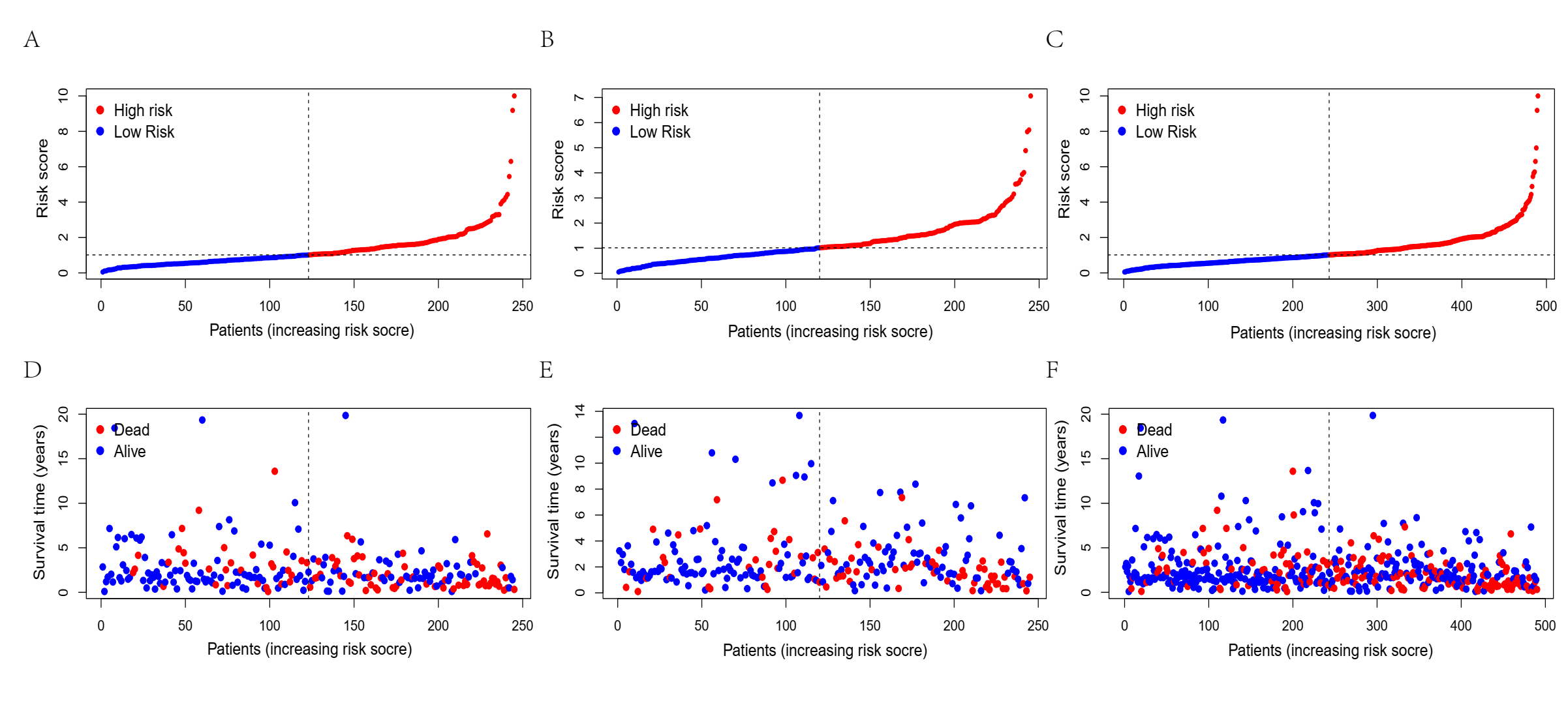


**Figure S2. The risk score model of the 6 NRlncRNAs signature.** A–C. Survival curves of NRlncRNAs model based on risk score of the train, test, and entire set, respectively. D-F. Distribution of risk score in train, test, and entire set.


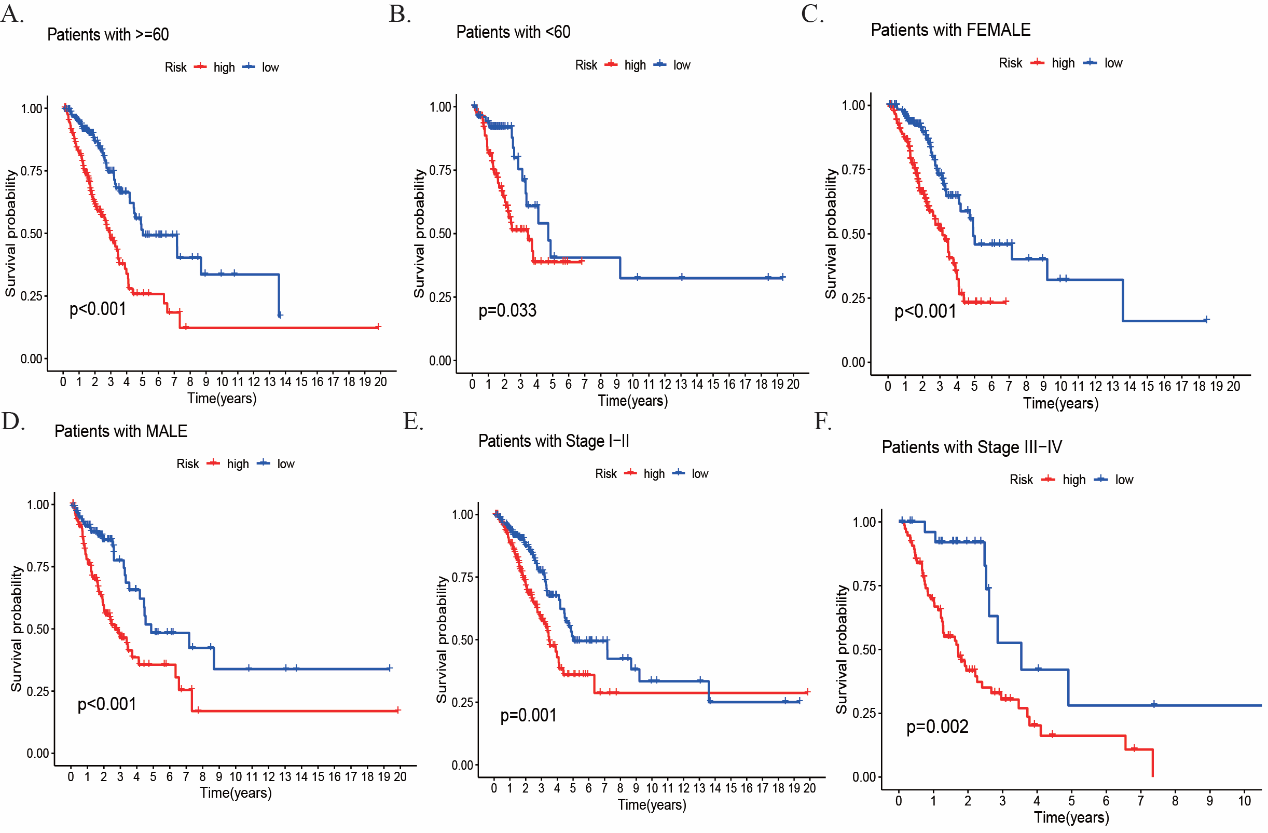


**Figure S3. The Kaplan-Meier curves of risk score stratified by age, gender, and stage.**


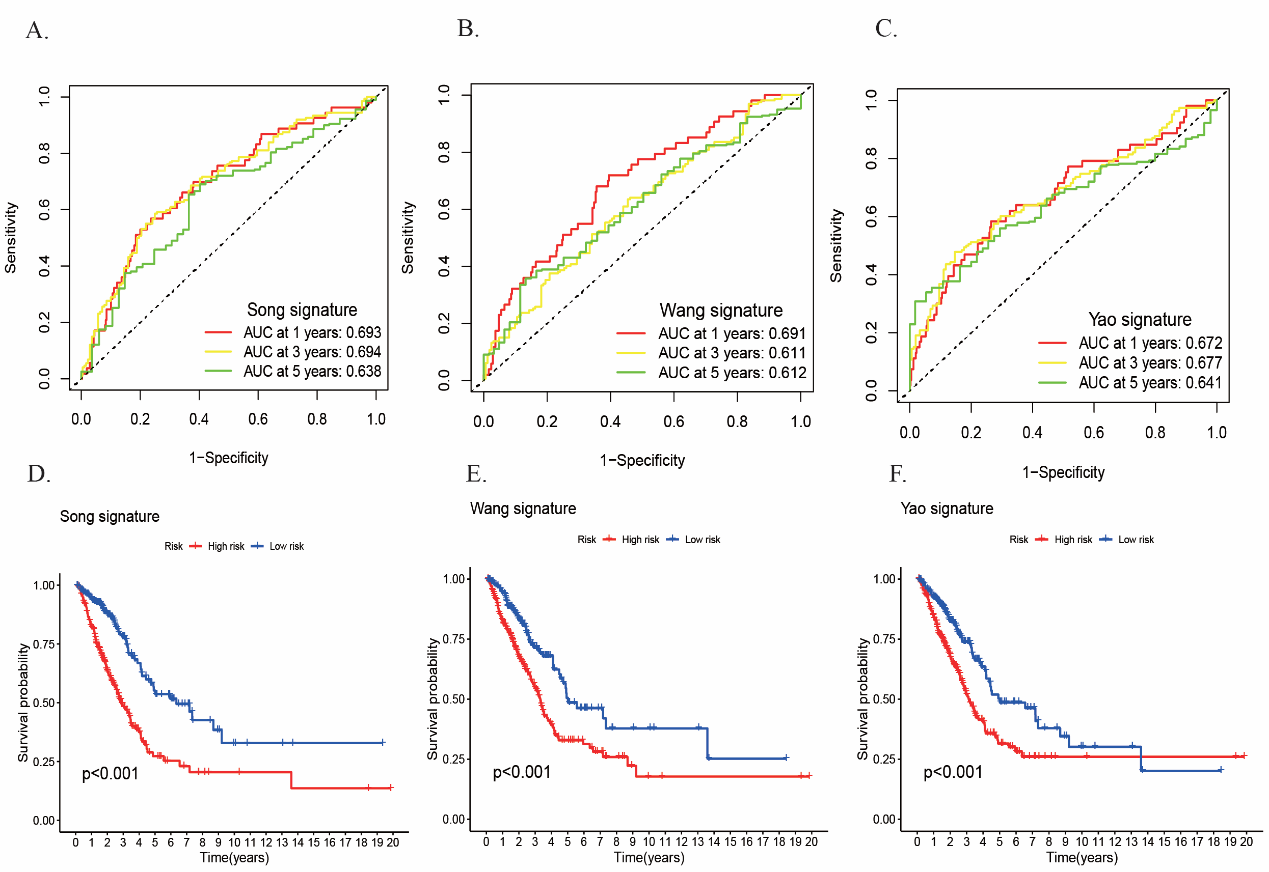


**Figure S4. Comparison of the survival predictive power of the signature and other risk model by ROC.**


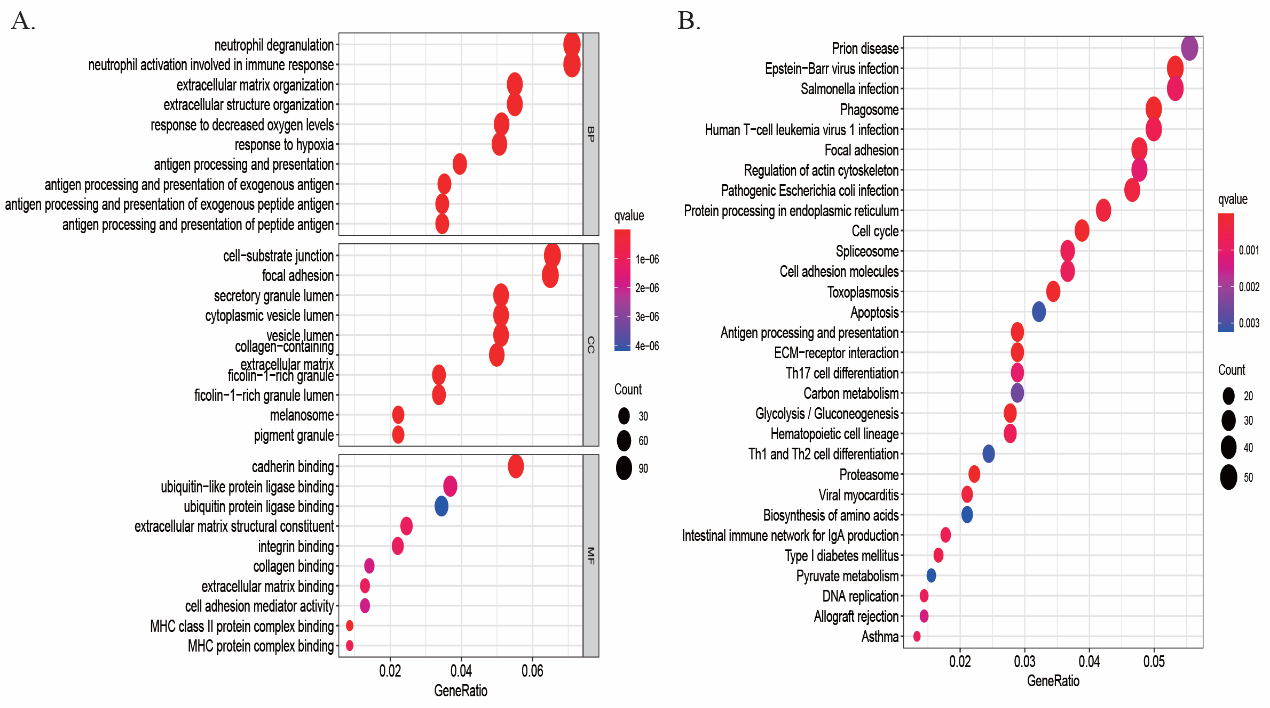
 **Figure S5. The function analysis of differentially expressed genes from high-and low-risk group.**


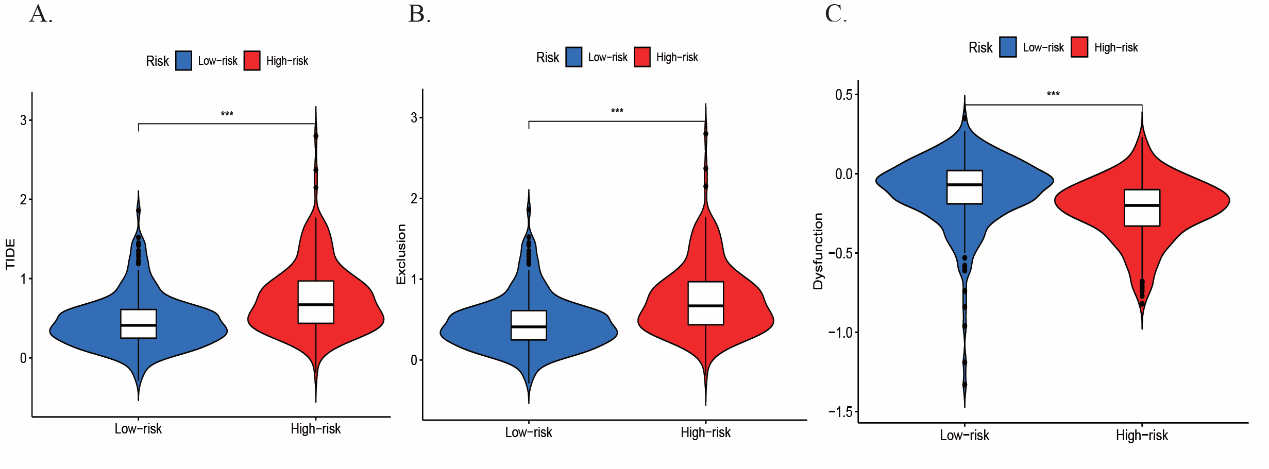


**Figure S6. The tumor immune dysfunction and exclusion analysis of prognostic NRGs-lncRNA signature.**


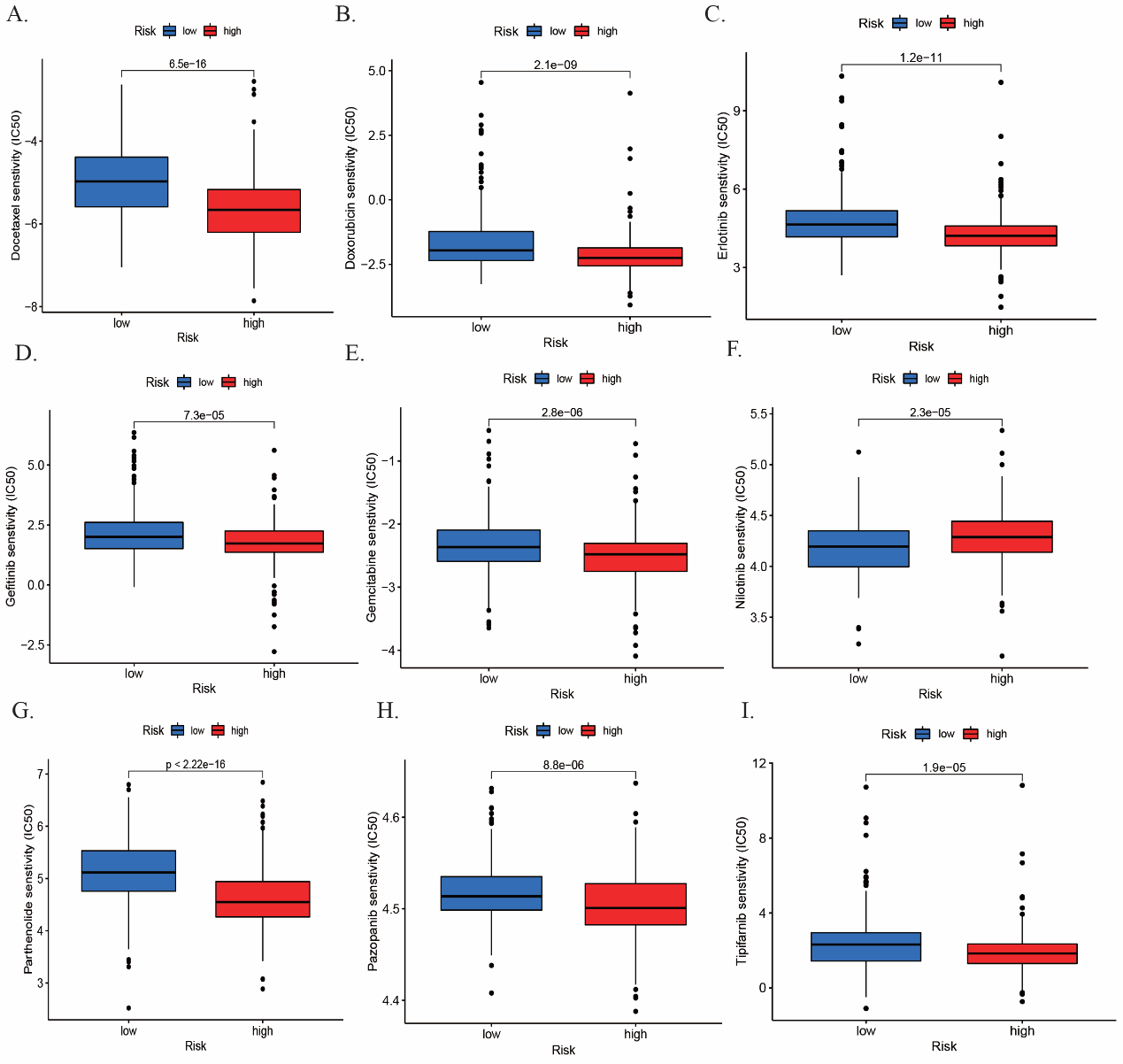


**Figure S7. The drug sensitivity of chemotherapy and target therapy of risk score in different risk group.**


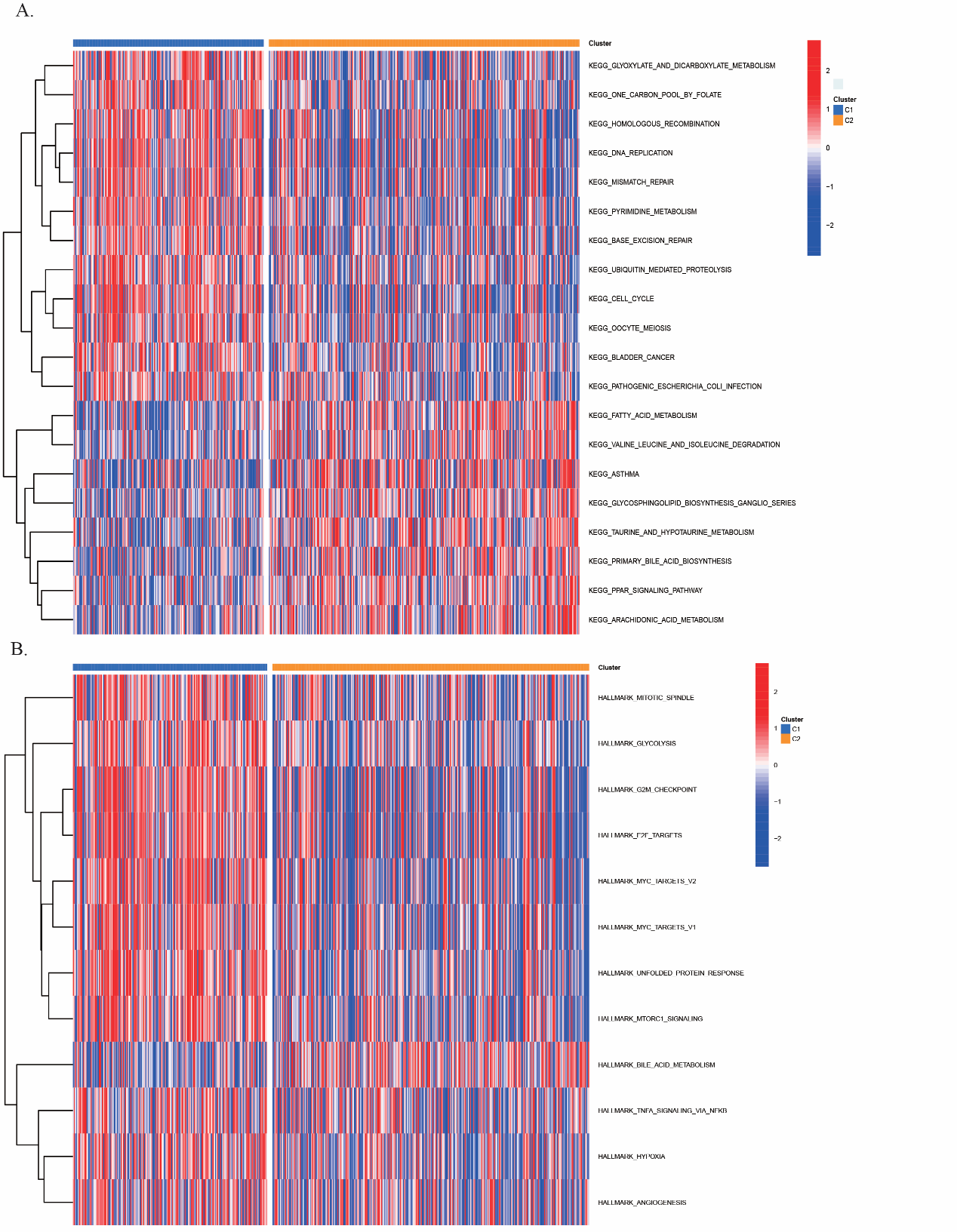


**Figure S8. GSVA enrichment analysis showing the activation states of biological pathways in the two distinct clusters.**


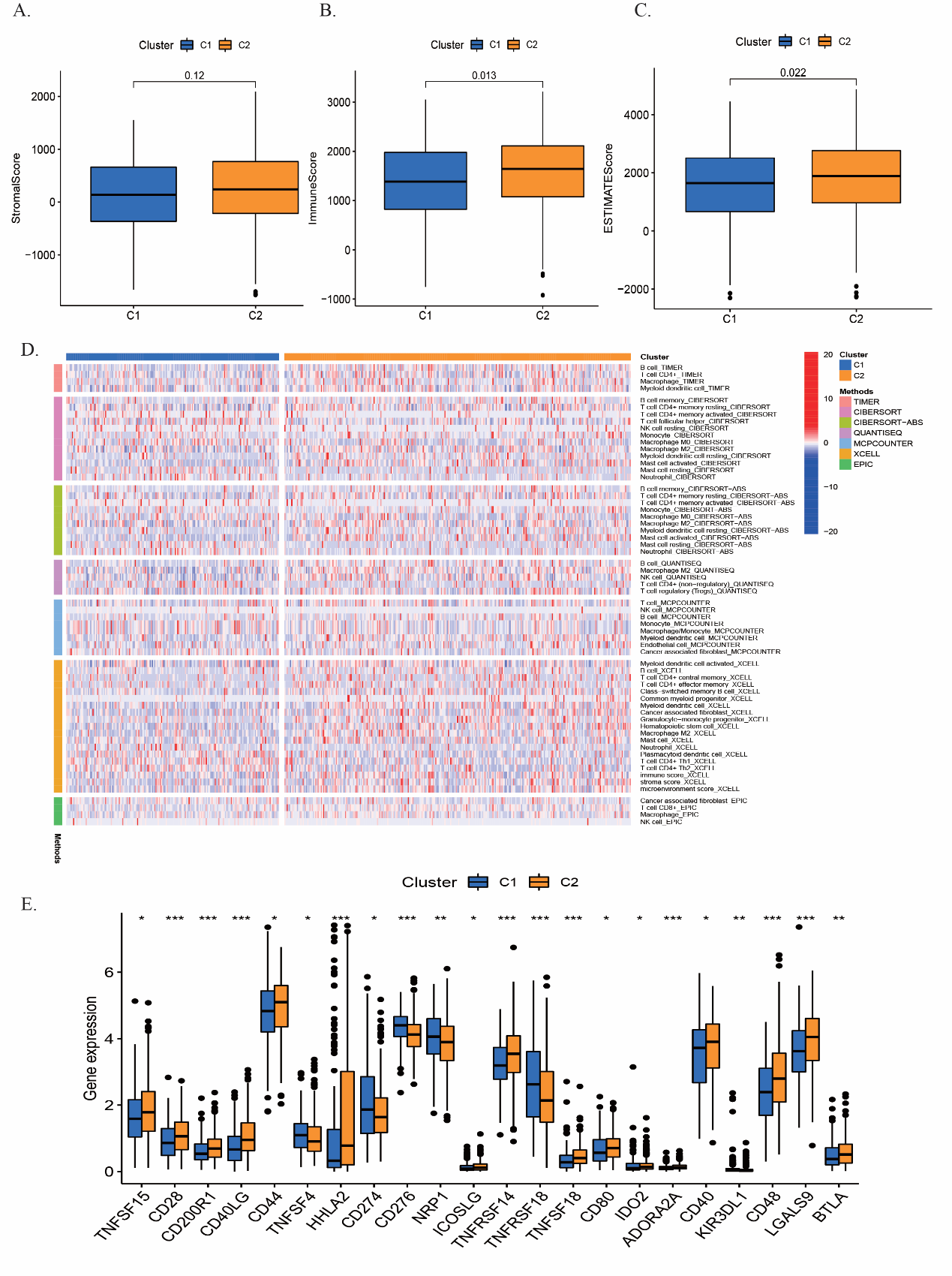


**Figure S9. Correlation of tumor immune cell microenvironment to two LUAD clusters.** A-C. The TME score (immune score, stromal score, and ESTIMATE score) in two clusters. D. The immune cell infiltration landscape between two clusters. E. The

differential expression level of immune checkpoint between two clusters.


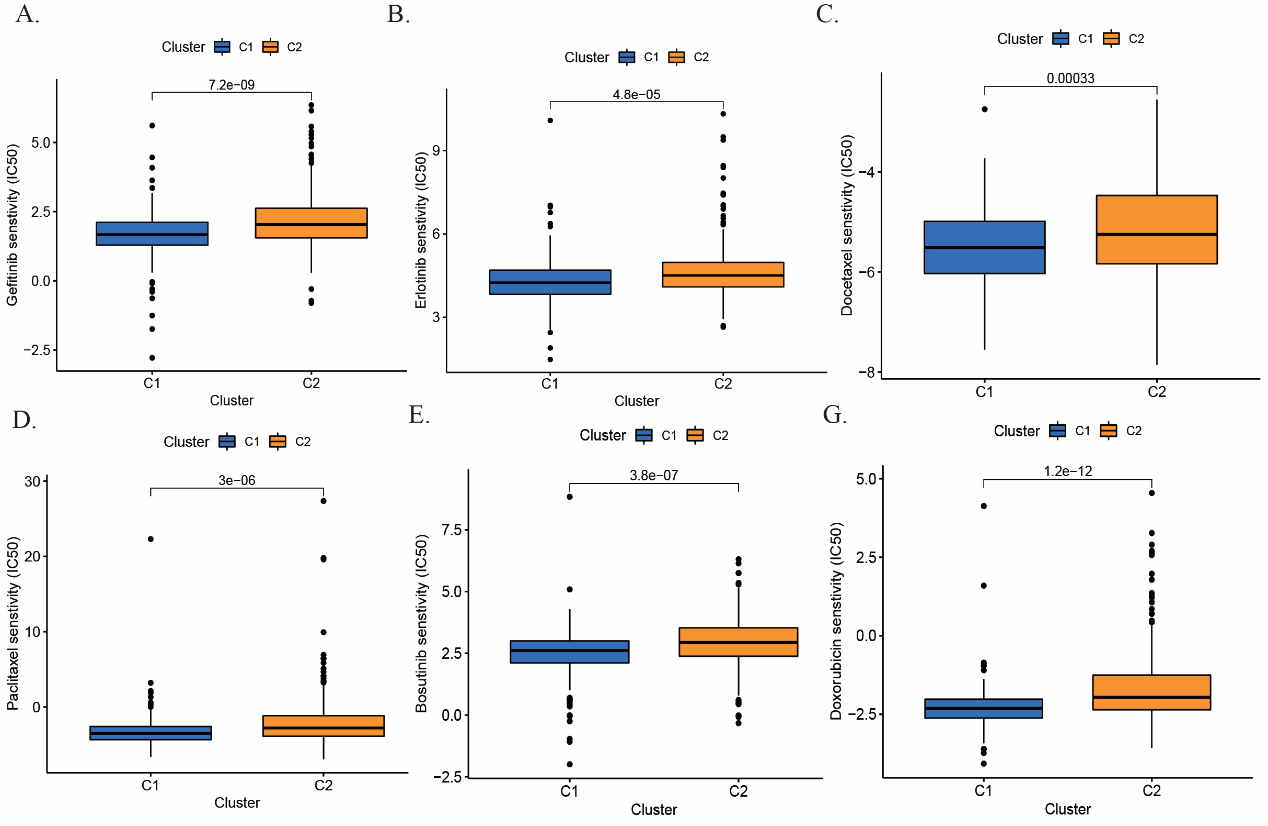


**Figure S10. Relationship between the clusters and sensitivity to chemotherapeutic and target therapy.**
